# Clinical Relevance of Nontuberculous Mycobacteria, Oman

**DOI:** 10.3201/eid1502.080977

**Published:** 2009-02

**Authors:** Sara H. Al-Mahruqi, Jakko van Ingen, Suleiman Al-Busaidy, Martin J. Boeree, Samiya Al-Zadjali, Arti Patel, P.N. Richard Dekhuijzen, Dick van Soolingen

**Affiliations:** Central Public Health Laboratory, Muscat, Oman (S.H. Al-Mahruqi, S. Al-Busaidy, S. Al-Zadjali, A. Patel); Radboud University Nijmegen Medical Centre, Nijmegen, the Netherlands (J. van Ingen, M.J. Boeree, P.N.R. Dekhuijzen); National Institute for Public Health and the Environment, Bilthoven, the Netherlands (J. van Ingen, D. van Soolingen)

**Keywords:** Atypical mycobacteria, nontuberculous mycobacteria, atypical mycobacteria infections, Mycobacterium avium complex, Mycobacterium simiae, Oman, dispatch

## Abstract

Little is known about the clinical relevance of nontuberculous mycobacteria (NTM) in the Arabian Peninsula. We assessed the prevalence and studied a random sample of isolates at a reference laboratory in Muscat, Oman. NTM cause disease in this region, and their prevalence has increased.

Nontuberculous mycobacteria (NTM) are common inhabitants of the environment and have been cultured from water, soil, and animal sources worldwide. NTM are opportunistic pathogens, mostly affecting patients with preexisting pulmonary disease such as chronic obstructive pulmonary disease or tuberculosis (TB), or those with systemic impairment of immunity. The latter group includes those with HIV infection, those using immunosuppressive drugs, and those with leukemia (*1*).

Because NTM are common in the environment and resistant to commonly used disinfectants, they can be present in nonsterile patient material such as sputum and contaminated medical equipment (bronchoscope washers or samples in the laboratory) and consequently cause pseudoinfection (*1*,*2*). To distinguish pseudoinfections from NTM disease and establish the clinical relevance of isolated NTM, the American Thoracic Society (ATS) has published diagnostic criteria (*1*).

Studies on the clinical relevance of NTM have traditionally been restricted to western countries, where the incidence of TB is relatively low. More recently, research has been initiated in African and East Asian countries (*1*–*5*). There have been no recent reports on isolation and clinical relevance of NTM on the Arabian Peninsula. We analyzed a random sample of NTM isolated from clinical samples in Oman by using molecular techniques. Their clinical relevance was retrospectively analyzed by applying the updated ATS diagnostic criteria for NTM disease (*1*).

## The Study

Prevalence of NTM at the Central Public Health Laboratory (CPHL), the national TB reference laboratory of the Ministry of Health in Muscat, Oman, was assessed by using a laboratory database. The CPHL Institutional Review Board reviewed and approved this study. The CPHL received 5,488 samples submitted for *Mycobacterium* spp. culture during 2006–2007. Samples were subcultured in Lowenstein-Jensen medium and automated Mycobacterial Growth Indicator Tubes (MGIT960; Becton Dickinson, Franklin Lakes, NJ, USA) and incubated at 36°C. A total of 491 (9%) samples yielded positive cultures. *M*. *tuberculosis* complex bacteria were isolated from 445 (91%) samples, and NTM were isolated from 46 samples (9%). Most NTM were cultured from respiratory samples (sputum, n = 36, 78%; bronchial lavage, n = 2, 4%); the remainder were from lymph nodes (n = 3, 6%), urine (n = 2, 4%), or other sources (n = 3, 6%). The percentage of NTM increased from 7.6% (18/235) in 2006 to 10.9% (28/256) in 2007.

Thirteen samples were randomly selected from all NTM isolated at CPHL during January 2006–September 2007. Selected NTM were subjected to molecular identification at the Dutch National Mycobacteria Reference Laboratory (National Institute for Public Health and the Environment, Bilthoven, the Netherlands). To identify NTM, after eliminating isolates of the *M*. *tuberculosis* complex by using the GenoType MTBC assay (Hain Lifesciences, Nehren, Germany), we used the Inno-Lipa Mycobacteria version 2 reverse line blot (Innogenetics, Ghent, Belgium). Both assays were used according to the manufacturer’s instructions. If no species-specific result was obtained, additional sequencing of the hypervariable region A of the 16S rDNA gene was performed (*6*).

The 13 samples were identified as 9 *M*. *avium* complex (MAC; 3 *M*. *intracellulare* sequevars, 3 *M*. *chimaera*, 1 *M*. *colombiense*, 1 *M*. *avium*, and 1 untyped), 1 *M*. *marinum*, 1 *M*. *kansasii*, 1 *M*. *simiae*, and 1 *M*. *flavescens* (Table). One sample could not be identified beyond the *M*. *avium*–*intracellulare*–*scrofulaceum* complex level because insufficient DNA was available for further analyses. Because our molecular identification method does not distinguish *M*. *marinum* from *M*. *ulcerans*, we performed a light exposure test, which identified yellow colony pigmentation typical for *M*. *marinum*. One sample yielded *M*. *tuberculosis* and *M*. *intracellulare* (Table).

**Table:** Clinical and microbiologic data for 13 patients with *Mycobacterium* spp. infections, Oman, 2006–2007*

Patient no./sex/ age, y	Species	AFB smear	Positive cultures	Source	Condition	Symp.	Chest radiograph	2007 ATS criteria	Therapy	Outcome
1/F/64	*M. intracellulare†*	+	Multiple	Sputum	–	PC, F, WL, CP, M	NP	Met	HRZE	Improved
2/M/29	MAIS complex	+	Single	Sputum	–	PC, Hp, WL	Cavities	Met	HRZE	Improved
3/F/31	*M. chimaera‡*	–	Multiple	Sputum	–	None	RUL bronch.	Not met	None	Stable
4/M/28	*M. chimaera‡*	+	Multiple	Sputum	–	PC, WL	RUL multiple scars, left lung destroyed, abscess, PT	Met	SClaCip	Failure
5/M/18	16S: *M*. *colombiense*	–	Single	BAL	HD	PC, Hp, CP	NP	Met	HRE	Improved
6/M/57	16S: *M*. *flavescens*	NP	Single	Urine	–	AP	NP	Not met	NA	NA
7/F/57	*M. simiae*	–	Single	Sputum	HIV	PC	Patchy opacities in LUL and lingula	Met	HRE	Improved
8/F/12	*M. kansasii* III/IV/V	–	Single	Sputum	–	None	Normal	Not met	NA	NA
9/M/43	*M. tuberculosis* and *M*. *intracellulare*†	+	Multiple	Sputum	–	PC, F, WL, M	Cavities	NA	HRZES	Failure
10/F/7	*M. marinum*	–	Single	Skin	–	Skin lesion	NP	Met	ECla	Improved
11/F/65	*M. chimaera‡*	+	Multiple	Sputum	–	PC, BA	Destroyed left lung	Met	ClaCip	Improved
12/F/83	*M. avium*	–	Single	Sputum	–	None	RUL infiltration	Not met	NA	NA
13/M/63	*M. intracellulare†*	+	Single	Sputum	Prior PNTM disease	PC, WL, M	Bronch., PI	Met	RECip	Improved

*AFB, acid-fast bacilli; Symp., symptoms; ATS, American Thoracic Society; PC, productive cough; F, fever; WL, weight loss; CP, chest pain; M, malaise/fatigue; NP, not performed; H, isoniazid; R, rifampicin; Z, pyrazinamide; E, ethambutol; MAIS, *M*. *avium*–*intracellulare*–*scrofulaceum*; Hp, hemoptysis; RUL, right upper lobe of lung; LUL, left upper lobe of lung; Bronch., bronchiectasis; PT, pleural thickening; S, streptomycin; Cla, clarithromycin; Cip, ciprofloxacin; 16S, identified by 16S rDNA gene sequencing; BAL, bronchoalveolar lavage fluid; HD, heart disease; AP, abdominal pain; NA, not applicable; BA, backache; PI, parenchymal infiltration; PNTM, pulmonary nontuberculous mycobacteria. †Reaction with the MIN-1 probe; *M. intracellulare* sequevar Min-A, -B, -C, or -D. ‡Reaction with the MIN-2 probe; *M. intracellulare* sequevar MAC-A, which was recently elevated to the species level (*M. chimaera*) (*7*).

We assessed the clinical relevance of isolates from 13 patients in a retrospective study by applying ATS diagnostic criteria and scoring demographic and clinical data. Results are detailed in the Table. Seven (54%) of the patients were female (mean age 43 years). Eight (62%) of 13 patients met the ATS diagnostic criteria and were thus likely to have NTM disease. Among 9 patients with MAC isolates, 6 (67%) had MAC disease. Most (11, 85%) isolates were cultured from pulmonary samples. Fibrocavitary and nodular-bronchiectatic pulmonary NTM disease were noted, with a predominance of fibrocavitary disease (Table).

Information on predisposing conditions was not available for most patients, although a destroyed lung on chest radiographs suggested previous pulmonary disease. One patient was HIV positive, and another patient had a relapse of pulmonary NTM disease.

Eight patients began treatment, mostly with first-line treatment for TB. Three patients with pulmonary MAC disease and 1 with *M*. *marinum* skin disease received regimens that included macrolides, fluoroquinolones, or both. Therapy resulted in clinical improvement in all but 1 patient. Most patients are still receiving treatment.

## Conclusions

A total of 9% all *Mycobacterium*-positive cultures at CPHL in Muscat, Oman, yielded NTM. Although this conclusion is based on limited data, the prevalence of NTM seems to be increasing in Oman. Few studies are available, but this increase may be true for the entire Middle East region.

Eight of 13 patients met the ATS diagnostic criteria; this finding probably reflects a selection bias because CPHL is a reference laboratory. Nevertheless, these findings indicate that NTM in Oman and throughout the Middle East region is a serious issue that requires attention by clinicians and microbiologists.

Most isolates were MAC members (9/13, 69%), a predominance also noted in previous studies in the United States, Europe, and west Asian countries (25%) (*1*,*8*), and South Korea (48%) (*9*). MAC isolates from Oman were mostly *M*. *intracellulare* sequevars. Infrequent isolation of *M*. *avium* is noteworthy, despite the small number of isolates in the random sample. Previous North American studies have suggested that *M*. *intracellulare* is the more common pulmonary pathogen within the MAC (*1*). We identified 3 pulmonary samples as MAC-A strains, which were recently elevated to species level as *M*. *chimaera* (*7*). Two samples were clinically relevant. Although *M*. *chimaera* has been assumed to be highly virulent (*7*), a recent study in Germany found only 3.3% of 90 *M*. *chimaera* isolates to be clinically relevant (*10*).

*M*. *colombiense* was first described as a causative agent of mostly disseminated disease in HIV patients from Colombia (*11*) and was recently isolated from a child with lymphadenopathy in Spain (*12*). Its isolation in other countries and from respiratory samples in HIV-negative patients has not been reported. Isolation of *M*. *simiae* is a serious concern because this species has been reported to be prevalent in the Middle East (*1*), and HIV-associated disease has been reported in the region (*13*).

Most patients in our study received standard treatment for TB. Although treatment should be prolonged and pyrazinamide discontinued because of natural resistance to pyrazinamide in NTM, the choice of first-line treatment for TB, without companion drugs such as macrolides or fluoroquinolones, is supported by a recently published trial of the British Thoracic Society (*14*).

In summary, NTM are a serious issue in Oman and their prevalence may be increasing. Our random sample demonstrates that MAC isolates are most frequently isolated. True NTM disease, on the basis of ATS diagnostic criteria, was diagnosed in 62% of the patients assessed. Isolation of NTM is clinically relevant on the Arabian Peninsula and warrants further study.

## References

[1] Griffith DE, Aksamit T, Brown-Elliot BA, Catanzaro A, Daley C, Gordin F, An official ATS/IDSA statement: diagnosis, treatment, and prevention of nontuberculous mycobacterial diseases. Am J Respir Crit Care Med. 2007;175:367–416. 10.1164/rccm.200604-571ST 17277290

[2] Portaels F. Epidemiology of mycobacterial diseases. Clin Dermatol. 1995;13:207–22. 10.1016/0738-081X(95)00004-Y 8521363

[3] Buijtels PC, Petit PL, Verbrugh HA, van Belkum A, van Soolingen D. Isolation of nontuberculous mycobacteria in Zambia: eight case reports. J Clin Microbiol. 2005;43:6020–6. 10.1128/JCM.43.12.6020-6026.2005 16333092 PMC1317173

[4] Chetchotisakd P, Kiertiburanakul S, Mootsikapun P, Assanasen S, Chaiwarith R, Anunnatsiri S. Disseminated nontuberculous mycobacterial infection in patients who are not infected with HIV in Thailand. Clin Infect Dis. 2007;45:421–7. 10.1086/520030 17638188

[5] Pettipher CA, Karstaedt AS, Hopley M. Prevalence and clinical manifestations of disseminated *Mycobacterium avium* complex infection in South Africans with acquired immunodeficiency syndrome. Clin Infect Dis. 2001;33:2068–71. 10.1086/323979 11698989

[6] Kirschner P, Springer B, Vogel U, Meier A, Wrede A, Kiekenbeck M, Genotypic identification of mycobacteria by nucleic acid sequence determination: report of a 2-year experience in a clinical laboratory. J Clin Microbiol. 1993;31:2882–9. 7505291 10.1128/jcm.31.11.2882-2889.1993 PMC266149

[7] Tortoli E, Rindi L, Garcia MJ, Chiaradonna P, Dei R, Garzelli C, Proposal to elevate the genetic variant MAC-A, included in the *Mycobacterium avium* complex, to species rank as *Mycobacterium chimaera* sp. nov. Int J Syst Evol Microbiol. 2004;54:1277–85. 10.1099/ijs.0.02777-0 15280303

[8] Martín-Casabona N, Bahrmand AR, Bennedsen J, Thomsen VØ, Curcio M, Fauville-Dufaux M, Non-tuberculous mycobacteria: patterns of isolation. A multi-country retrospective survey. Int J Tuberc Lung Dis. 2004;8:1186–93. 15527150

[9] Koh WJ, Kwon OJ, Jeon K, Kim TS, Lee KS, Park YK, Clinical significance of nontuberculous mycobacteria isolated from respiratory specimens in Korea. Chest. 2006;129:341–8. 10.1378/chest.129.2.341 16478850

[10] Schweickert B, Goldenberg O, Richter E, Göbel UB, Petrich A, Buchholz P, Occurrence and clinical relevance of *Mycobacterium chimaera* sp. nov., Germany. Emerg Infect Dis. 2008;14:1443–6. 10.3201/eid1409.071032 18760016 PMC2603105

[11] Murcia MI, Tortoli E, Menendez MC, Palenque E, Garcia MJ. *Mycobacterium colombiense* sp. nov., a novel member of the *Mycobacterium avium* complex and description of MAC-X as a new ITS genetic variant. Int J Syst Evol Microbiol. 2006;56:2049–54. 10.1099/ijs.0.64190-0 16957098

[12] Esparcia O, Navarro F, Quer M, Coll P. A case of lymphadenopathy caused by *Mycobacterium colombiense.* J Clin Microbiol. 2008;46:1885–7. 10.1128/JCM.01441-07 18305134 PMC2395067

[13] Al-Abdely HM, Revankar SG, Graybill JR. Disseminated *Mycobacterium simiae* infection in patients with AIDS. J Infect. 2000;41:143–7. 10.1053/jinf.2000.0700 11023758

[14] Jenkins PA, Campbell IA, Banks J, Gelder CM, Prescott RJ, Smith AP. Clarithromycin vs ciprofloxacin as adjuncts to rifampicin and ethambutol in the treatment of opportunist mycobacterial pulmonary diseases and an assessment of the value of immunotherapy with *Mycobacterium vaccae*: a pragmatic, randomised trial by The British Thoracic Society. Thorax. 2008;63:627–34. 10.1136/thx.2007.087999 18250184

